# A Book about Doctors

**Published:** 1861-07

**Authors:** 


### NORTH AMERICAN

### MEDICO-CHIRURGICAL REVIEW.

JULY, 1861.

### Analytical and Critical Reviews.

ART. I.—*A Book about Doctors.* By J. CORDY JEAFFRESON, author of "Novels and Novelists," "Crewe Rise," etc. etc. Reprinted from the English Edition. New York: Rudd & Carleton. London: Hurst & Blackett, 1861. 12mo., pp. 490.

THE "Book" by Mr. Jeaffreson is written in a light, sketchy, and superficial vein, without regard to method—although it is divided into chapters—and without any attempt to delineate the inner or esthetic life of the profession, in the which, as not being himself a member of it, he could not be supposed to enter. The author, in collecting "the best of those medical Ana that have been preserved by tradition or literature, has not only done his best to combine or to classify old stories, but, also, cautiously to select his materials, so that his work, while affording amusement in the leisure hours of doctors learned in their craft, might contain no line that should render it unfit for the drawing-room table." In carrying out this intention, many *bon mots*, jokes and ridiculous incidents, the coarseness of which would not be redeemed by their wit, must of necessity have been omitted. Although many of the anecdotes now recorded have long formed part of the gossip of our medical literature, there is not a little in these pages that is new and curious. The author takes pain to acknowledge the friendly aid which he received, in the progress of his work, from Dr. Munk, the learned librarian of the College of Physicians, (London,) and from Dr. Diamond, of Twickenham House.

Interspersed between chapters on great names in the medical profession are others on "Sticks," and "Wigs," and "Quacks," and "Fees;" "The Generosity and Parsimony of Physicians," "The Quarrels of Physicians," and "The Loves of Physicians." The collection is large and various, but it is made up of such incongruous materials that the reader will find it difficult to acquire any very definite opinions of the real character and merits of the medical profession. But this is not what the author aimed at. He looked to writing a readable, and, on the whole, an amusing book, and he has succeeded. If his publishers are in good humor, and the crowd of readers pleased, what need he care about neglect of the canons of taste, or the censures of the logical critic?

Touching "sticks," attention should be drawn, the author tells us, to the *rabdos*, so respected in every chapter of the world's history—

"To the fasces of the Roman lictors, which every schoolboy honors (often unconsciously) with an allusion, when he says he will *lick*, or vows he won't be *licked*—to the herald's staff of Hermes, the caduceus of Mercury, the wand of Æsculapius, and the rods of Moses and the contending sorcerers—to the mystic bundle of nine twigs, in honor of the nine muses, that Dr. Busby loved to wield, and which many a simple English parent believes Solomon, in all his glory, recommended as an element in domestic jurisdiction—to the sacred wands of savage tribes, the staffs of our constables and sheriffs, and the highly-polished gold sticks and black rods that hover about the ante-rooms of courts at St. James or Portsoken. The rule of thumb has been said to be the government of this world; and what is this thumb but a short stick, a *sceptre*, emblematic of a sovereign authority, which none dares to dispute? 'The stick,' says the Egyptian proverb, 'came down from heaven.'"

But the only sticks about which the author cares to speak, on the present occasion, are physicians' canes, barbers' poles, and the twigs of rue which are still strewn on a ledge before the prisoner in the dock of a criminal court. The physician's cane, a very ancient part of his insignia, has, like the three-tailed wig, disappeared within the last century. Time was when no doctor of medicine would have ventured to pay a professional visit, or even to be seen in public, without this mystic wand and the oracular head-gear. The celebrated gold-headed cane which Radcliffe, Mead, Askew, Pitcairn, and Baillie successively bore, is preserved in the College of Physicians, bearing the arms of those gentlemen. In one respect it deviated from the physician's cane proper, in its having a cross-bar, almost like a crook; whereas a physician's wand ought to have a knob at the top. We discoursed, in former numbers of this journal, of the adventures of "the gold-headed cane," in connection with the portraits and character of those who were in succession its possessors. The knob of the physician's cane, "in olden times, was hollow, and contained a vinaigrette, which the man of science always held to his nose when he approached a sick person, so that its fumes might protect him from the noxious exhalations of his patient."

The custom of sprinkling aromatic herbs before the prisoners, when brought into court for trial, arose from the abominable state of the prisons, so that the poor creatures dragged from them and placed in the dock were frequently in such an offensive condition that the noxious effluvia from their bodies made even seasoned criminal lawyers turn pale. From the same cause came the chaplain's bouquet, with which that reverend officer was always provided when accompanying a criminal to Tyburn.

The complete separation of the insignia of the surgeons and barbers dates but little, if at all, beyond the present century. Lord Thurlow, in a speech delivered in the House of Peers on the 17th of July, 1797, opposing the surgeons' incorporation bill, said that, "by a statute still in force, the barbers and surgeons were each to use a pole. The barbers were to have theirs blue and white, striped, with no other appendage; but the surgeons', which was the same in other respects, was, likewise, to have a gallipot and a red rag, to denote the particular nature of their vocation." The mistake of the chancellor, in this description, is corrected by the author, who says that the chirurgical pole, properly tricked out, ought to have a line of blue paint, another of red, and a third of white, winding round its length, in a regular serpentine progression,—the blue representing the venous blood, the more brilliant color the arterial, and the white thread being symbolic of the bandage used in tying up the arm after withdrawing the ligature.

"Possibly, in ancient times, the physician's cane and the surgeon's club were used more actively. For many centuries fustigation was believed in, as a sovereign remedy for bodily ailments as well as moral failings, and a beating was prescribed for an ague as frequently as for picking and stealing. This process Antonius Musa employed to cure Octavius Augustus of sciatica. Thomas Campanella believed that it had the same effect as colocynth administered internally. Galen recommended it as a means of fattening people. Gordinus prescribed it in certain cases of nervous irritability: 'Si sit juvenis, et non vult obedire, flagelletur frequenter et fortiter.' In some rural districts ignorant mothers still flog the feet of their children, to cure them of chilblains. And there remains on record a case in which club-tincture produced excellent results on a young patient to whom Desault gave a liberal dose of it."

Next to his cane, the physician's wig was the most important of his accoutrements. Our readers may have read of allusions to the dress doctorial in olden times, but few of them have seen so minute a description of the received costume as in the following lines:—

"Each son of Sol, to make him look more big,
Had on a large, grave, decent, three-tailed wig;
His clothes half-trimmed, with button-holes behind,
Stiff were the skirts, with buckram stoutly lined;
The cloth-cut velvet or more reverend black,
Full made, and powdered half-way down his back;

Large decent cuffs, which near the ground did reach,
With half-a-dozen buttons fixed on each.
Grave were their faces; fixed in solemn state,
These men struck awe, their children carried weight:
In rev'rend wigs old heads young shoulders bore,
And twenty-five or thirty seemed threescore."

The last wig worn by a doctor, off the stage, was by Dalmahoy, who, with the title of colonel, was celebrated as a vender of drugs and nostrums of all sorts—sweetmeats, washes for the complexion, scented oil for the hair, pomades, love-drops, and charms.

The last of the silk-coated physicians, who held on to the old costume, was Dr. Henry Revell Reynolds, one of the physicians who was in attendance on George III., during his long and melancholy affliction—insanity.

"Though this gentleman came quite down to living times, he persisted to the end in wearing the costume—of a well-powdered wig, silk coat, breeches, stockings, buckled shoes, gold-headed cane, and lace ruffles—with which he commenced his career. He was the Brummell of the Faculty, and retained his fondness for delicate apparel to the last. Even in his grave-clothes the coxcombical tastes of the man exhibited themselves. His very cerements were of 'a good make.'

'Here well-dressed Reynolds lies,
As great a beau as ever;
We may, perhaps, see one as wise,
But sure a smarter—never!'"

Changes in the mode of physicians visiting their patients were gone through in England nearly similar to those which have occurred in our American cities. At first, visits were made on horseback, and subsequently, in a carriage; but, in London, it was the fashion for doctors to sit sideways on their horses and in foot-clothes like women.

"In our own day an equipage of some sort is considered so necessary an appendage to a medical practitioner, that a physician without a carriage (or a fly that can pass muster for one) is looked at with suspicion. He is marked down *mauvais sujet*, in the same list with clergymen without duty, barristers without chambers, and gentlemen whose Irish tenantry obstinately refuse to keep them supplied with money."

The author closes his notice of the cane, wig, silk coat, stockings, side-saddle, and carriage of the old physician, with referring to the muff, in which, in cold weather, his fingers and forearms were incased. We confess to a fondness for the muff which, in our severe winters, would be not only a luxury but a positively healthy portion of dress, that ought not to be monopolized by the other sex.

The chapter on "Early English Physicians," in the "Book" before us, abounds in curious retrospective lore. Among the more celebrated and learned of the class under notice, was Dr. John Phreas, who was born about the beginning of the fifteenth century and educated at Oxford, where he obtained a fellowship on the foundation of Baliol College. Italy was then and for a long period afterward the chief seat of medical learning, and to that country went a number of the most eminent English physicians to complete their studies and acquire a knowledge of anatomy and clinical medicine not procurable at home. Phreas obtained his degree at Padua, and in that section of the peninsula he gained a large fortune by the practice of his profession. He was a poet and an accomplished scholar. Some of his epistles, in MS., are still preserved in the Baliol library and at the Bodleian. The gift of a bishopric from Pope Paul II., for his translation of Diodorus Siculus, cost him his life. "A disappointed candidate for the same preferment is said to have poisoned him before the day appointed for his consecration."

Dr. Thomas Linacre, one of the chief founders and the first president of the London College of Physicians, was a learned doctor, both in medicine and theology; and physician successively to Henry VII., Henry VIII., Edward VI., and the Princess Mary; thus exhibiting in his own person a conjunction of honors rarely conferred on any individual, however learned and estimable he may have been. So distinguished a man merits the introduction here of a few particulars not found in Mr. Jeaffreson's pages. Linacre was the first Englishman who read Aristotle and Galen in the original Greek. On his travels in Italy, he was received with marked distinction at Florence by Lorenzo de Medici; he studied eloquence at Bologna under Politian, one of the most elegant Latinists in Europe. On his return to England, among other occupations, he taught the Princess Catharine the Italian language, and wrote a treatise on mathematics, and another on grammar, the latter of which was pronounced by Melancthon to be inferior to none of its kind then extant. His pure Latinity elicited the praises of his friend Erasmus, who said of him, that he was "*vir non exacti tamen sed severi judicii.*"* The scriptural readings of Linacre sometimes led him to conclusions which were not indicative of his being a very profound theologian. He read, for the first time, shortly before his death, the New Testament; and after having gone over the 5th, 6th, and 7th chapters of St. Matthew, he threw the book away, and swore that this was either not the Gospel, or we were not Christians. Without pretending to justify the impatient criticism of Linacre, we are bound to confess that the sermon on the mount by our Saviour, as found in the chapters just named, is not the code which governs the conduct of

* The Gold-Headed Cane, pp. 80–3. By Dr. Macmichael.

all professing Christians. A fling at one's neighbor's misdeeds or at his doctrinal errors is too often made at the expense of the mildness, meekness, and self-abasement which theoretically are supposed to belong to the religious censor.

Linacre bequeathed his house, that in which the College of Physicians first met, to this corporation. On this house the physician's arms, granted by Christopher Barker, Garter King-at-Arms, Sept. 20, 1546, may still be seen. The motive for Linacre taking holy orders, before his death, is not known. "Possibly he imagined the sacerdotal garb would be a secure and comfortable clothing in the grave." In quoting this suggestion of the author, we must come in for a share of the stricture on its flippancy.

In the generation next succeeding that of Linacre, was John Kaye, or Key—Caius he has long been somewhat pedantically named. Like his predecessor, Caius is associated with letters not less than with medicine. He was first heard of as reading lectures on Aristotle in the University of Padua—an unexpected post of honor to be enjoyed by an Englishman. He procured an act of incorporation for Gonvil College, Cambridge University, with the additional title of Caius, and endowed it afterward with considerable estates for the maintenance of an additional number of fellows and scholars. Caius was Fellow, Censor, and President of the (London) College of Physicians. He died, 1573, in the sixty-third year of his age. On his tomb, in his college, and constructed under his own direction, was inscribed the following brief epitaph: "*Fui Caius. Vivit post funera virtus.* Obiit 1573, Æt. 63."

The year of the death of Caius was that of the birth of Mayerne, who became a baron of France and a knight of England. He was a lucky courtier if not a skillful physician.

"After leaving France and permanently fixing himself in England, he kept up his connection with the French, so that the list of his monarch patients may be said to comprise two French and three English sovereigns: Henry IV. and Louis XIII. of France, and James I., Charles I., and Charles II. of England. Sir Theodore Mayerne died at Chelsea, in the eighty-second year of his age, on the 15th of March, 1665."

His library went to the College of Physicians, and his wealth to his only daughter, who was married to the Marquis of Montpouvillon. Mayerne was one who, not long ago, would have been called a heroic practitioner. Calomel was habitually prescribed by him in scruple doses; sugar of lead he mixed largely in his conserves. His sweetest compound was his "Balsam of Batts," strongly recommended as an unguent for hypochondriacal persons, into which entered adders, bats, sucking whelps, earth-worms, hog's grease, the marrow of a stag, and the thigh-bone of an ox.

A greater man comes before us in the person of Harvey, who died two years after Mayerne, or in 1667. Aubrey says of the discoverer of the circulation of the blood :—

"He was not tall but of the lowest stature, round-faced, olivaster (like waintscott) complexion, little eie—round, very black, full of spirit; his haire was black as a raven, but quite white twenty years before he dyed. I remember he was wont to drink coffee, which he and his brother Eliah did, before coffee-houses were in fashion in London. He was, as all the rest of his brothers, very cholerique; and in his younger days wore a dagger (as the fashion then was;) but this doctor would be apt to draw out his dagger upon very slight occasion. He rode *on horseback, with a foot-cloath, to visit his patients, his man following on foot, as the fashion then was, was very decent, now quite discontinued.*"

The most noted of the physicians of the Elizabethian age was William Bulleyn, related to the unfortunate Anna Boleyn, the frail and beautiful wife of Henry VIII. Mr. Jeaffreson speaks in high terms of the acuteness and learning displayed in the numerous works of William Bulleyn, "which are among the most interesting prose writings of the Elizabethian era." In his "Book of Simples," the doctor discourses pleasantly on the virtues of different herbs as well as on the more choice productions of the garden. "Grapes are spoken of as cultivated and brought to a high state of perfection in Suffolk and other parts of the country. Hemp is humorously called gallow grasse, or neck weed." Bulleyn was a bold and courageous surgeon. "Soft chirurgians," he used to say, "make foul sores." He recommends the surgeon to have a gladsome countenance, so as to reassure the patient. His recipes for "an embrocation," "a good emplaster," and "*electuarium de gemmis*," are curiosities in their way. Did room permit, we would transcribe them. Among many wonderful properties of the electuarium, it causeth kings and noblemen to be bold-spirited, the body to smell well, and it engendereth to the face good color.

Apropos of this, the author introduces the following prescription which an unfortunate physician wrote for a nervous lady :—

"℞.—Great Western, 350 shares;<br>

Eastern Counties }<br>

North Middlesex } āā 1050.<br>

M. ft. Haust. i. Om. noc. cap.

"This direction to a delicate gentlewoman to swallow nightly two thousand four hundred and fifty railway shares was regarded as evidence of the physician's insanity, and the management of his private affairs was forthwith taken out of his hands. But assuredly it was as rational a prescription as Bulleyn's Electuarium Gemmis."

In the prescriptions of this physician which most excite one's risibility is "a smal yong mouse rosted," for the cure of a child suffering under a nervous malady. By many this would be preferred to a preparation of snails, recommended on the same authority. "Snayles broken from the shelles and sodden in whyte wyne, with oyle and sugar, are very holsome because they be hwat and moist for the straightness of the lungs and cold cough. Snails stamped with camphory and leven will draw forth prycks in the flesh."

Among the most famed physicians of the seventeenth century was Sir Thomas Browne, whose writings are now deservedly classical. The chief of these are "Religio Medici," and "Pseudoxia Epidemica," a treatise on vulgar errors. After taking his degrees of B.A. and M.A. in Oxford, he traveled over different parts of Europe, and obtained his degree of doctor of medicine at Leyden.

"Returning to England, he settled at Norwich, married a rich and beautiful Norfolk lady named Mileham, and for the rest of his days resided in that ancient city, industriously occupied with an extensive practice, the pursuits of literature, and the education of his children. When Charles II. visited Norwich in 1671, Thomas Browne, M.D., was knighted by the royal hand."

The eminent physician, traveled and accomplished, and who had seen much of the world, was, notwithstanding, diffident to shyness. As was his manner, so was his dress. "In his habit of cloathing, he had an aversion to all finery, and affected plainness both in fashion and ornaments."

The "Religio Medici" created an unprecedented sensation at the time of its appearance, but it shocked the orthodox by its free and searching inquiries, which, in their eyes, amounted to infidelity, while it was open, at the same time, to the charge of scientific heresies.

Sir Kenelm Digby is introduced by the author as one of the adverse critics of the "Religio Medici." He finds a place in the list of professional worthies, as an amateur healer of wounds by his celebrated Sympathetic Powder, which consisted of blue vitriol dissolved, again crystallized, and pulverized, and exposed for a length of time to a summer sun. "It cured wounds in the following manner: if any piece of a wounded person's apparel having on it the stain of blood that had proceeded from the wound was dipped in water holding in solution some of this sympathetic powder, the wound of the injured person would forthwith commence a healing process. It mattered not how far distant the sufferer was from the scene of operations, or whether he was conscious of them." Cases were gravely related by Sir Kenelm and others of the efficacy of this powder. What absurdity was ever yet palmed on public credulity that was not bolstered up by cases and certificates! "For a time the sympathetic powder was very generally believed in, and it doubtless did as much good as harm, by inducing people to throw from their wounds the abominable messes of grease and irritants which were then honored by the name of plasters."

The inventor of this wonderful powder is evidently a favorite with Mr. Jeaffreson, who lavishes on him a string of epithets, as "the eccentric, gallant, brave, credulous, persevering, frivolous, Sir Kenelm Digby. A Mecænas, a Sir Philip Sydney, a Dr. Dee, a Beau Fielding, and a Dr. Kitchener all in one, this man is chief of these extravagant characters that astonish the world at rare intervals, and are found nowhere except in actual life. No novelist of the most advanced section of the idealistic school would dare to create such a personage as Sir Kenelm." From the period when he was knighted, after his return from attending Charles I., (then Prince of Wales,) at Madrid, he was before the world as courtier, cook, lover, warrior, alchemist, gentleman of the bedchamber, and commissioner of the navy. "It is difficult to say to which he was most devoted—his king, his church, literature, or his beautiful and frail wife, Venetia Stanley, whose charms fascinated the many admirers on whom she distributed her favors, and gained her Sir Kenelm for a husband when she was the discarded mistress of Richard, Earl of Dorset. She had borne the earl children; so his lordship, on parting, settled on her an annuity of five hundred pounds ($2500) *per annum.*"

"The lives of three physicians—Sydenham, Sir Hans Sloane, and Heberden—completely bridge over the uncertain period between old empiricism and modern science." On the signal merits of Sydenham as a medical reformer, by bringing physicians back to a careful observation of nature, we are not called upon here to expatiate, nor to explain his meaning when he told Sir Richard Blackmore, in reply to a question as to the best course for a medical student to pursue: 'Read Don Quixote; it is a very good book—I read it still.'"

Sir Hans Sloane was "an Irishman by birth and a Scotchman by descent; he exhibited in no ordinary degree the energy and politeness of either of the sister countries. After a childhood of extreme delicacy, he came to England and devoted himself to medical study and scientific investigation." Among other distinctions won by him, he was made President of the College of Physicians in 1719, and President of the Royal Society, (on the death of Sir Isaac Newton,) in 1727, and he was appointed physician to George II. In addition to these honors, he was the first medical practitioner advanced to the dignity of a baronetcy.

"On the death of Sir Hans Sloane, on the 11th of January, 1753, his museum and library passed into the hands of the nation for a comparatively small sum of money, and became the nucleus of our British Museum." Sir Hans had two medical successors in the presidency of the Royal Society—Sir John Pringle, Bart., elected November 30, 1772, and William Hyde Wollaston, M.D., elected June 29, 1820.

A chapter on "The Apothecaries and Sir Samuel Garth" contains sundry particulars on the College of Apothecaries, and the differences between this body and the College of Physicians. The outlines of the latter were given by us in a former number of this journal, (March, 1860,) introductory to a review of the Medical Institutions of Philadelphia. The first charter obtained in the fourth year of James I. was for the incorporation into one body of the Grocers and Apothecaries of the City of London. In the long and angry disputes between the physicians and apothecaries, Pope took the side of the former. "Like Johnson, Parr, and all men of enlightenment and sound scholarship, he had a high opinion of the faculty." To Dr. Arbuthnot he wrote :—

> "Friend to my life, which did not you prolong
> The world had wanted many an idle song."

His habitual tone, when speaking of the medical profession, was that of warm admiration and affection. In the "Imitations of Horace," he says :—

> "Weak though I am of limb, and short of sight,
> Far from a lynx and not a giant quite,
> I'll do what Mead and Cheselden advise,
> To keep these limbs and to preserve these eyes."

"It is true that he elsewhere ridicules Mead's fondness for rare books, and Sloane's passion for butterflies; but at the close of his days he wrote, in a confidential letter to a friend of the faculty, 'They are, in general, the most amiable companions and the best friends, as well as the most learned men I know.'"

In speaking of "the origin of the memorable Dispensarian Campaign between the College of Physicians and the Company of Apothecarries," Mr. Jeaffreson makes a remark which is, we fear, not without its application to some of our own cities. "Half the peddling little charitable institutions, dispensaries or hospitals, that at the present time rob the rich and do harm to the poor in every quarter of London, originated in 'the friends' of young physicians and surgeons conspiring together to get them 'the position of being attached to a hospital staff.'"

The name of Garth is better known in the annals of literature than of medicine. In his poem, "The Dispensary," the apothecaries and anti-dispensarians were covered with ridicule. The dispensarians was the name given to the physicians of the college who established a dispensary where medicines were vended to the poor at cost price. Other members of the college were opposed to this measure, and hence were called anti-dispensarians. With these latter the apothecaries combined, and they agreed not to recommend the dispensarians. Garth, full of jest and amiability, did more to create merriment at the Kit-Kat Club than either Swift or Arbuthnot.

"His practice was a good one, but his numerous patients prized his *bon mots* more than his prescriptions. His enemies averred that he was not only an epicure, but a profligate voluptuary and an infidel; Pope, however, wrote of him after his death: 'If ever there was a good Christian without knowing himself to be so, it was Dr. Garth.'"

It must ever be borne in mind, in favor of Garth's claims on posterity, that to him Dryden owed honorable interment.

"When the great poet died, Garth caused his body to be conveyed to the College of Physicians, and started a public subscription to defray the expenses of the funeral. He pronounced an oration over the deceased at the College in Warwick Lane, and then accompanied it to Westminster Abbey."

Many are the stories preserved of Garth's social humor. The one, of his postscript to a letter to a friend, in which he says that he would write more, but for a d—d tall, impudent Irishman looking over his shoulder all the time, and the denial of the fact roared out by the man, has found its way into Joe Miller's jest-book. Another is less known: "Stumbling into a Presbyterian church, one Sunday, for pastime, he found a pathetic preacher shedding tears over the iniquity of the earth. 'What makes the man greet [weep]?' asked Garth of a by-stander. 'By my faith,' was the answer, 'and you too would greet, if you were in his place and had as little to say.' 'Come along, my dear fellow,' responded Garth to his new acquaintance, 'and dine with me. You are too good a fellow to be here.'"

Another of his repartees is worth repeating:—

"At the Kit-Kat he once stayed to drink long after he had said he must be off to see his patients. Sir Richard, more humane than the physician, or possibly, like the rest of the world, not disinclined to be virtuous at another's expense, observed: 'Really you ought to have no more wine, but be off to see those poor devils.'

"'It's no great matter,' Garth replied, 'whether I see them to-night or not; for nine of them have such bad constitutions, that all the physicians in the world can't save them; and the other six have such good constitutions, that all the physicians in the world can't kill them.'"

Apropos of "Quacks," which form the subject of the sixth chapter of the work before us, we may remark that the profession is making advances in medical ethics as it is in scientific discovery and applications. We find here recorded some instances of quackery in eminent medical men of the last century which would compromise the standing of any respectable physician of the present day. An outline history of the progress of quackery and the deportment and arts of the quack is pleasantly told by the author.

"Queen Anne's weak eyes caused her to pass from one empiric to another for the relief they all promised to give, and in some cases even persuaded that they gave her. She had a passion for quack oculists; and happy was the advertising scoundrel who gained her majesty's favor with a new collyrium. For, of course, if the greatest personage in the land said that Professor Bugalo was a wonderful man, a master of his art, and inspired by God to heal the sick, there was no appeal from so eminent an authority. How should an elderly lady, with a crown on her head, be mistaken? Do we not hear the same arguments every day in our own enlightened generation, when the new chiropodist, or rubber, or inventor of a specific for consumption, points to the social distinction of his dupes as conclusive evidence that he is neither supported by vulgar ignorance nor afraid to meet the most searching scrutiny of the educated? Good Queen Anne was so charmed with two of the knaves who by turns enjoyed her countenance, that she had them sworn in as her own oculists in ordinary, and one of them she was even so silly as to knight. This lucky gentleman was William Reade, originally a botching tailor, and to the last a very ignorant man, as his 'Short and Exact Account of all the Diseases incident to the Eyes' attests; yet he rose to the honor of knighthood and the most lucrative and fashionable physician's practice of his period. Surely, every dog has his day." "It is true that Sir William Reade was unable to read the book which he had written (by the hand of an amanuensis)."

"Queen Anne's other 'sworn oculist,' as he and Reade termed themselves, was Roger Grant, a cobbler and Anabaptist preacher. He was a prodigiously vain man, even for a quack, and had his likeness engraved on copper." "This man, according to the custom of his class, was in the habit of publishing circumstantial and minute accounts of his cures. Of course his statements were a tissue of untruths, with just the faintest possible admixture of what was not altogether falsehood."

The author enumerates, among "the medical quacks" of the last century, the Rev. John Hancocke, D.D. This gentleman should rather be called a clerical quack, who published a small tract in 1723, a copy of which is in our possession, entitled "*Febrifugum Magnum;* or Common Water the best Cure for Fevers, and probably for the Plague." The history of empiricism would present little to revolt common sense and sound ethics, were its pages to include no worse features than those displayed in the "*Febrifugum Magnum.*" No concealment of the article used is attempted, no gain results to the propounder, and each person is left to make a trial on or by himself of a liquid which is, avowedly, indispensable to health, and a powerful auxiliary, if not a primary agent, in the cure of disease. In a more restricted range, we might make the same remark of good Bishop Berkeley's "Tar Water," which at one time enjoyed so much vogue as a panacea.*

---

* The reader, desirous of learning the history and therapeutical applications of the use of common water as recommended by Hancocke, and of tar water as praised by Berkeley, will be gratified by a reference to Dr. Bell's volume on "Baths," &c.

The belief in the Loutherbourg miracles, performed by a man and his wife of that name, was a good specimen of the credulity of the latter part of the eighteenth century. These impious creatures proclaimed that God had endowed them with a miraculous power of healing the impoverished sick, by looking upon and touching them. The dupes to these pretensions were among all classes. "Quacks have, in all ages, found staunch supporters among the powerful and the affluent."

"Dumoulin, the physician, observed at his death, that 'he left behind him two great physicians, Regimen and River Water.' A due appreciation of the truth embodied in this remark, coupled with that masterly assurance, without which the human family is not to be fleeced, enabled the French quack Villars to do good to others and to himself at the same time. This man, in 1723, confided to his friends that his uncle, who had recently been killed by an accident at the advanced age of one hundred years, had bequeathed to him the recipe for a nostrum which would prolong the life of one who used it to a hundred and fifty, provided only that the rules of sobriety were never transgressed. Whenever a funeral passed him in the street, he said aloud: 'Ah! if that unfortunate creature had taken my nostrum, he might be carrying that coffin, instead of being carried in it.' This nostrum was composed of nitre and Seine water, and was sold at the ridiculously cheap rate of five francs [a dollar] a bottle. Those who bought it were directed to drink it at certain stated periods, and, also, to lead regular lives, to eat moderately, drink temperately, take plenty of bodily exercise, go to and rise from bed early, and to avoid mental anxiety. In an enormous majority of cases the patient was either cured or benefited."

The Abbé Pons declared Villars to be the superior of the marshal of the same name. "The latter," said he, "kills men, the former prolongs their existence." But Villars's secret finally leaked out; and his patients deserted him, with probably less wisdom than they manifested in coming to him.

Quite an amusing chapter is taken up with the sayings and doings, whims and oddities of the celebrated Dr. Radcliffe. The most prominent of these were brought to the notice of our readers in our department "The Editor's Table," of this journal for 1860. Radcliffe exhibited a compound of meanness of disposition in money matters on a small scale, and many instances of considerate liberality to those in distress. His poor relations had, indeed, no share of this liberality during his lifetime; but his posthumous fame, in this particular, is established on an immovable basis, by his munificent bequests to the University of Oxford, in the founding and endowment of the Radcliffe Library, the Radcliffe Observatory, the Radcliffe Infirmary, and the Radcliffe Traveling Fellowships. What a shining example for any of our rich physicians of the present age, who happen to be burdened with a large fortune and have no children! Radcliffe provided liberally for his poor relations after his death.

"Radcliffe, the Jacobite partisan, the physician without learning, and the luxurious *bon-vivant*, who grudged the odd sixpence of his tavern scores, was born at Wakefield, in Yorkshire, in the year 1650. His extraction was humble, his father being only a well-to-do yeoman." "But, though Radcliffe was a plebeian, he contrived, by his shrewd humor, arrogant simplicity, and immeasurable insolence to hold both Whigs and Tories in his grasp. The two factions of the aristocracy bowed before him; the Tories from affection to a zealous adherent of regal absolutism, and the Whigs from a superstitious belief in his remedial skill, and a fear that in their hours of need he would leave them to the advances of death."

In the chapter headed "The Doctor as a *Bon-vivant*," we learn that "the bottle and the board were once the doctor's two favorite companions. More than one eminent physician died in testifying his affection for them. In the days of tippling they were the most persevering of tavern-haunters. No wonder that some of them were as fat as Daniel Lambert, and that even more died sudden deaths from apoplexy. The obesity of Dr. Stafford was celebrated in an epitaph:—

'Take heed, O, good traveler, and do not tread hard,
For here lies Dr. Stafford, in all this church-yard.'

"Dr. Beddoes was so stout that the Clifton ladies used to call him their 'walking feather bed.' Dr. Flemyng weighed twenty stone and eleven pounds, till he reduced his weight by abstinence from the delicacies of the table, and by taking a quarter of an ounce of Castile soap every night.

"Dr. Cheyne's weight was thirty-two stone, till he cured himself by persevering in a temperate diet. Laughing at two unwieldy noblemen, whose corpulence was the favorite jest of all the wits in the court, Louis XV. said to one of them, 'I suppose you take little or no exercise.' 'Your majesty will pardon me,' replied the bulky duke, 'for I generally walk two or three times round my cousin every morning.'"

The physicians of the seventeenth and eighteenth centuries, if they did not lead, certainly put no restraints, either by precept or example, on the intemperance prevailing among all classes. Tobias Walker and John Archer, both physicians to Charles II., were representative men of their class. The first was a strenuous advocate for the free use of wine, which he calls the physic that does not dull, but sets a true edge upon nature, and after operation leaveth no venomous contact. "John Archer, the author of 'Every Man his own Doctor,' and 'Secrets Disclosed,' was an advocate of generous diet and enlightened sensuality." Like Whitaker, he did not think it beneath him to keep a number of apothecaries' shops and to live by the sale of drugs as well as fees. His cordial diet drink was advertised as costing 2*s.* 6*d.* (50 cents) per quart; for a box containing 30 morbus pills, the charge was 5*s.*; 40 corroborating pills were to be had for the same sum. He recommended his patients to smoke, and he sold tobacco, of a quality superior to the ordinary articles of commerce, at 2*s.* and 1*s.* an ounce.

"Whilst Whitaker and Archer were advising men to smoke and drink, another physician of the court was inventing a stomach-brush, in some respects much like the bottle-brush with which fly poison ought to be taken from the interior of black bottles before wine is committed to them. This instrument was pushed down the gullet, and then poked about and turned round, much in the same way as a chimney-sweeper's brush is handled by a dextrous operator on soot. It was recommended that gentlemen should thus sweep out their insides not oftener than once a week, but not less frequently than once a month. What a race of men our ancestors must have been! The curious may find not only a detailed description, but an engraved likeness of this remarkable stomach-brush, in the *Gentleman's Magazine*, vol. xx., for the year 1750."

Was this instrument ever really used in the manner just described? We are ourselves skeptical on this point.

Freind, the historian of Medicine, continually visited his patients in a state of intoxication. Gibbons—the "Nurse Gibbons," as he was contemptuously called by Radcliffe—did something for English dinner-tables worth remembering.

"He brought into domestic use the mahogany, with which one has so many pleasant associations. His brother, a West India captain, brought over some of the wood as ballast, thinking it might possibly turn to use. At first the carpenters, in a truly conservative spirit, refused to have anything to do with the 'new wood,' saying it was too hard for their tools. Dr. Gibbons, however, had first a candle-box and bureau made for Mrs. Gibbons out of the condemned material. The bureau so pleased his friends, among whom was the Duchess of Buckingham, that her grace ordered a similar piece of furniture, and so introduced the wood into high life, where it quickly became the fashion."

In addition to the names already given, we find recorded in this volume those of Dr. George Fordyce, Beauford, and Barrowby, as *bon-vivants*, of whom various characteristic anecdotes are told. Sir Richard Jebb is introduced here, in a forced connection, as careless about humoring the stomach, in his dietetic advice to his patients. This physician was celebrated for his abrupt and harsh replies to his patients, some of whom were not backward in retorting on him for his rudeness. "That's my way," said he to a noble invalid, astonished at his rudeness. "Then," answered the sick man, pointing to the door, "I beg you will make that your way." Even to the king Sir Richard was plain spoken. George the Third lamented to him the restless spirit of his cousin, Dr. John Jebb, the dissenting minister. "And, please your majesty," was the answer, "if my cousin were in heaven, he would be a reformer."

The chapter, for the most part devoted to the feasting of physicians, is concluded with a few words on their fasts. The most abstinent collection of medical men was that of a scientific club that used to meet in the house of a grocer in the Strand, London. The principal members were Drs.

Heberden, Turton, Geo. Baker, Sir John Pringle, and Sir Wm. Watson, and Lord C. Cavendish, who officiated as president. "Each member paid sixpence per evening for the use of the grocer's dining-room. The club took in one newspaper, and the only refreshment allowed to be taken at the place of meeting was—water." We have been accustomed to speak frequently of the sumptuary regulations of the old medical club of a limited number of Philadelphia physicians, which confine the refreshments of the evening entirely to tea and coffee, with bread and butter and a few light cakes. It has been in existence for nearly thirty years, and of its eight original members, who had been editors of the North American Medical and Surgical Journal, all are still living.

"Fees" make an amusing chapter in the "Book," and furnish matter for many extracts. It is not long since we touched on the subject,* and, partly on this account and partly owing to our rapidly waning space, we must forego, in a great measure, the present temptation. The contrasted moods of a patient, when sick and suffering, and looking to his doctor as the saviour of his life, and when convalescent and in recovered health, are well hit off by the author.

"Illustrative of the same truth is a story told of Bouvart. On entering one morning the chamber of a French marquis, whom he had attended through a very dangerous illness, he was accosted by his noble patient in the following terms: 'Good-day to you, M. Bouvart; I feel quite in spirits, and think my fever has left me.' 'I am sure it has,' replied Bouvart, dryly. 'The very first expression you used convinced me of it.' 'Pray, explain yourself.' 'Nothing is easier. In the first days of your illness, when your life was in danger, I was your *dearest friend;* as you began to grow better I was your *good Bouvart;* and now I am M. Bouvart; depend upon it, you are quite recovered.'"

"Sir," said Sir Theodore Mayerne to a friend whose proffered fee he, unexpectedly to the latter, had pocketed, "I made my will this morning, and if it should appear that I refused a fee, I might be deemed *non compos.*" The author does justice to Abernethy in denying the authenticity of many of the stories told of this eminent surgeon, as illustrative of his brusque and even rude speech and ways. On all proper occasions he was considerate and kind, and was very careful not to take fees from patients if he suspected them to be in indigent circumstances. "Side by side with stories of the benevolence of the faculty frequent anecdotes of their stinginess might be told." As we have not room at this time to record their liberal doings, we shall be readily excused for omitting to repeat instances of their reverse conduct, believing firmly, moreover, that these latter are exceptional to the general practice of physicians in receiving honorariums from their patients.

---

* North American Medico-Chirurgical Review, November, 1859.

We only notice the chapter on "Bleeding" to record our earnest protest against the following sentence, as mischievously inaccurate: "Blood-letting," says Mr. Jeaffreson, "so long a popular remedy with physicians, has, like tobacco-smoking for medicinal purposes, fallen into disuse and contempt." We confidently affirm that no physician or surgeon who is well read in clinical annals, and a calm and experienced observer of disease, will adopt such a sweeping conclusion. True science acknowledges no such vagaries of fashion, no such jumping from one extreme to another.

We are fortunately prevented from what we should otherwise feel the necessity of passing our prescribed limits by following the author in his chapter on Dr. Richard Meade, the learned, the lucky, the urbane, the tasteful and the munificent physician, who was the contemporary of Radcliffe and Freind. His personal traits and professional character and liberality have been sketched in our pages, *apropos* of our notice of "the Gold-headed Cane."* It was truly observed by Dr. Johnson, that "Dr. Meade lived more in the broad sunshine of life than almost any man." This eminent physician had, however, his quarrels, the most bitter of which was that with Woodward, Professor of Physic at the Gresham College, by whom he was bitterly attacked in the "State of Physic and Diseases." On one occasion some insult offered by Woodward so infuriated Mead, that the latter drew his sword and called on his adversary to defend himself. The duel terminated in Mead's favor, as far as martial prowess was concerned, for he disarmed Woodward and ordered him to beg his life. "Never, till I am your patient!" was the happy answer of Woodward.

At another time Mead had to run, if not for his life at least to save his peruke and himself from a good scratching from the hands of Sarah, Duchess of Marlborough, who became infuriated at a remark which the physician had dared to make when visiting the sick Duke.

"Mead was not the first of his name to enter the medical profession. William George Meade was an eminent physician at Tunbridge Wells; and, dying there on the 4th of November, 1652, was buried at Ware, in Hertfordshire. This gentleman left £5 ($25) a year forever to the poor; but he is more remarkable for longevity than generosity. He died at the extraordinary age of 148 years 9 months. This is one of the most astonishing instances of longevity on record. Old Parr, dying at 152 years, exceeded it only by 4 years. The celebrated Countess of Desmond was some years more than 140 at the time of her death. Henry Read, minister of Hardwicke, Northampton County, numbered only 132 years; and the Lancashire woman (*the Cricket of the Hedge*) did not outlive her 141st year. But all these ages become insignificant when put

* North American Medico-Chirurgical Review, March, 1860.

by the side of 169 years, to which Henry Jenkins protracted his earthly sojourn."

"The imagination as a remedial power," is a fine theme for narrative and commentary. "And if the delusions of talismans, amulets and chains, and the impostures of Mesmer, have had no greater consequences, they have at least afforded to the observant and reflective much valuable instruction with regard to the constitution of the human mind."

"Red objects had a mysterious influence on inflammatory diseases; and yellow ones had a similar power on those who were discolored with jaundice. Edward II.'s physician, John of Gaddesden, informs us: 'When the son of the renowned King of England lay sick of the small-pox, I took care that everything round the bed should be of a red color, which succeeded so completely that the Prince was restored to perfect health without a vestige of a pustule remaining.' Even as late as 1765 this was put in practice to the Emperor Francis I."

In England, more than in any other region of Christendom, the superstition of the miraculous power over disease by the royal touch found supporters.

"From Edward the Confessor, down to Queen Anne, who laid her healing hands on Samuel Johnson, it flourished; and he was a rash man who, trusting to the blind guidance of human reason, dared to question that divinity which encircles kingship. Doubtless the gift of money made to each person who was touched did not tend to bring the cure into disesteem. It can be easily credited that, out of the multitude who flocked to the presence of Elizabeth and the Stuart kings for the benefit of their miraculous manipulations, there were many shrewd vagabonds who had more faith in the coin than in the touch bestowed upon them. The majority were, however, as sincere victims of delusion as those who, at the close of the last century, believed in the efficacy of metallic tractors, and those who now unconsciously expose their intellectual infirmity as advocates of electro-biology and spirit-rapping. The populace, as a body, unhesitatingly believed that their sovereigns possessed this faculty as the anointed of the Lord. A story is told of a Papist who, much to his astonishment, was cured of the king's evil by Elizabeth, after her final rupture with the Court of Rome.

"'Now I perceive,' cried the man, 'by plain experience, that the excommunication against the Queen is of no effect; since God hath blessed her with such a gift.'"

Montaigne and Burton are quoted on the power of the imagination; each of these writers treating the subject after his quaint and peculiar fashion. The most notable forerunner of Mesmer in England was Valentine Greatrakes, who, in Charles the Second's reign, performed several "marvaillous cures by the stroaking of the hands."

"Doubtless many of Valentine's patients were suffering, not under scrofulous affections, but comparatively innocent tumors—for his cures were rapid, complete, and numerous. A second impulse gave him the power of curing ague, and a third inspiration of celestial aura imparted to him command, under certain conditions, over all human diseases. His modes of operation were various. When an afflicted person was laid before him, he usually offered up a prayer to God to help him to make him the humble instrument of Divine mercy; and, invariably, when a patient derived benefit from his treatment, he exhorted him to offer up his thanks to his Heavenly Father. After the initiatory supplication the operator passed his hands over the affected part of the sick person's body, sometimes over the skin itself, and sometimes over the clothes. The manipulations varied in muscular force from delicate tickling to violent rubbing, according to the nature of the evil spirits by which the diseased people were tormented. Greatrakes's theory of disease was the scriptural one—the morbific power was a devil, which had to be expelled from the frame in which it had taken shelter. Sometimes the demon was exorcised by a few gentle passes; occasionally it fled like a well-bred dog, at the verbal command of the physician, or retreated on being gazed at through the eyes of the mortal it tormented; but frequently the victory was not gained till the healer rubbed himself—like the rubber who, in our own days, makes a large income at Brighton—into a red face and copious perspiration."

The success of Greatrakes was signal, and certificates of the most marvelous cures were not withheld; but his triumph was of short duration.

"His professions were made the butts of ridicule, to which his presence of mind and volubility were unable to respond with effect. It was asserted by his enemies that his system was only a cloak under which he offended the delicacy of virtuous women, and roused the vile passions of the unchaste. His tone of conversation was represented as compounded of the blasphemy of the religious enthusiast and the blasphemy of the obscene profligate. His boast, that he never received a fee for his professional services, was met by a flat contradiction, and a statement that he received presents to the amount of £100 ($500) at a time from a single individual. This last accusation was never clearly disposed of; but it is probable that the reward he sought (if he looked to any) was restoration, through court influence, to the commission of magistrate for the county, and the lost clerkship of the peace."

Next among curative wonders was Perkin's metallic tractors, which enjoyed uncommon vogue, until it was shown by Dr. Haygarth that two pieces of wood, properly shaped and painted, produced the same miraculous effects on the uninitiated sick. Few individuals have ever gained the celebrity won in a short time by Mesmer, who, without originating a single idea, contrived to accumulate vast wealth and fix his name on a branch of psychological science. Following out the notions of some who had preceded him, he first used steel magnets as a means of cure, when applied externally. Afterward he threw these aside and produced still greater marvels by the employment of his fingers and eyes. His audacity was unequaled. He told Dr. Egg von Ellekon, as a reason why he preferred river to spring water, that the former is exposed to the sun's rays, and is in consequence magnetized, and that he himself, to insure a steady supply from the sun, had magnetized this planet twenty years before. He had charge of the husband of the celebrated school-mistress, Madame Campan, for inflammation of the lungs. His first prescription of an old empty bottle to be put by the side of the patient proving ineffectual,* he seized the occasion of Madame Campan's temporary absence to bleed and blister the sick man—a treatment which proved more efficacious. "But imagine Madame Campan's astonishment, when, on her husband's recovery, Mesmer asked for and obtained from him a written certificate that he had been cured by Mesmerism!" Our contempt for the knave, on such an occasion, must be extended in full measure to the willing dupe who in part makes himself accessory to the fraud thus practiced on the community. As far as related to any scientific pretensions set up in favor of Mesmerism, they were shown to be without any foundation by a Committee of the French Academy, in whose labors Franklin, then in France, participated. Mesmer died in obscurity on the 5th of March, 1815.

In the chapter entitled "Make Way for the Ladies," although not a lady appears, Mr. Jeaffreson says: "Of the many doctresses who have flourished in England during the last two hundred years, only a few have left any memorial of their actions behind them." The most successful of the wise women of the last century was Mrs. Joanna Stephens, who claimed and obtained a grant of £5000 ($25,000) from the British Parliament for a secret remedy which was represented to be a certain cure for the stone. The chief medicines used were calcined egg-shells, snails, soap, and a decoction of certain herbs. "Five thousand pounds for such stuff as this! and the time was coming when the nation grudged an inadequate reward to Jenner, and haggled about the purchase of Hunter's Museum!!"

But a more remarkable case of feminine success in the doctoring line was that of Mrs. Mapp, better known as "Crazy Sally, of Epsom," who was a contemporary of Mrs. Stephens. "She was an enormous fat, ugly, drunken woman, known as a hunter of fairs, about which she loved to reel, screaming and abusive, in a state of roaring intoxication." This attractive lady was a bone-setter, daughter of a man named Wallin, of the same craft, and so much esteemed was she for skill in her art, that the town of Epsom offered her £100 if she would reside there for a year. Crazy Sally awoke one morning and found herself famous. Patients of rank and wealth flocked in from every quarter. In an evil hour she mar-

* He advised, in the first instance, as the only way to cure M. Campan, "to lay in his bed by his side, one of three things—a young woman of brown complexion, a black hen, or an old bottle." Madame C., of course, preferred the trial of the bottle for her husband.

ried an Epsom swain and became Mrs. Mapp. Her husband lived with her only a fortnight, during which short space of time he thrashed her soundly twice or thrice, and then decamped with a hundred guineas of her earnings. Mrs. Mapp continued to reside in Epsom, but she visited London once a week. Her journeys to and from the metropolis were performed in a chariot drawn by four horses, with servants wearing splendid liveries. She won the confidence of Sir Hans Sloane to such a degree as to induce him to put his niece under her care, for curvature of the spine, or, to use the scientific language of the newspapers, "whose back had been broken nine years, and stuck out two inches." The circumstances of Mrs. Mapp sitting one evening at the Lincoln's-Inn-Fields theatre, between Taylor, the quack oculist, and Ward, the dry-salter, furnishes one of those *apropos*, which Mr. Jeaffreson so readily snatches at, to an account of this celebrated Chevalier Taylor—"a cunning, plausible, shameless blackguard. Such being his character, of course he was successful in his vocation of quack." Dr. King, in his "Anecdotes of his own Times," speaks of him in the following more laudatory strain:—"No charlatan ever appeared with fitter and more excellent talents, or to greater advantage; he has a good person, is a natural orator, and has a faculty of learning foreign languages. He has traveled over all Europe, and has always with him an equipage suitable to a man of the first quality, and has been introduced to most of the sovereign princes, from whom he has received many marks of their liberality and esteem." Taylor's autobiographic sketch of himself, in which he speaks of his loves and adventures, is a strange medley of rhapsody, boasting, and doubtless a large addition of lies. He used to sign himself "The Chevalier Taylor, Ophthalmiater, Pontifical, Imperial, and Royal." The chevalier had a son, also an oculist and a quack, who is represented in the page before us to be "an illiterate, vulgar, and licentious scoundrel." What shall be thought of posthumous praise when we are told that this man was honored, after his death, with a long memoir in the *Gentleman's Magazine*, as one "whose philanthropy was exalted so fully as to class him with a Hanway or a Howard?"

We shall not follow the author in his chapter on "Messenger Monsey," a coarse and vulgar, yet learned physician, who has left nothing behind him worth recording, if we except some *bon mots* not always fit for ears polite, and caustic repartees uttered with a design to wound and offend.

Culling from three chapters headed "Pedagogues turned Doctors," "Akenside," and "Literature and Art," we shall be able to collect most of the scattered notices in them relating to physicians who attained to more or less celebrity as poets. First among them, in the order of time, although classed among the pedagogues by Mr. Jeaffreson, is Sir Richard

Blackmore. The son of a Wiltshire attorney, he was educated at Westminster and Oxford, residing in all thirteen years in the University. On leaving Oxford, he passed through a course of searching poverty, and became a schoolmaster. This did not prevent him from traveling over most of the then most accessible countries on the continent, and taking his doctor's degree in the University of Padua. The author commits an anachronism in making Sir Richard consult Dr. Heberden as to what authors he should read, and in giving as the answer of the latter, "Don Quixote." The question was asked by Blackmore of Sydenham, and answered as above. The slip is the more noticeable as the true version had been given on a preceding page, (57.)

"As a poet, Blackmore failed; but as a physician he was for many years one of the most successful men in his profession." "Honest, industrious, honorable, and cordially liked by his personal friends, he was by no means the paltry fellow that Dryden and Pope represented him to be. Johnson, in his brilliant memoir, treated him very unfairly, and clearly was annoyed that his conscience would not allow him to treat him worse." "A sincerely religious man, Blackmore was offended with the gross licentiousness of the drama and all those productions of the poets which constituted the light literature of the eighteenth century. To his eternal honor, Blackmore was the first man who had the courage to raise his voice against the evil, and give utterance to a manly indignation at the insults offered nightly in every theatre to public decency." "Blackmore made the fatal error of writing too much. His long poems wearied the patience of those who sympathized with his goodness of intention."

We have already spoken of Garth as poet and physician. The next most illustrious example of this union is found in the person of Mark Akenside, a man of "most excellent contradictory parts."

"There were two Akensides—Akenside the poet, and Akenside the man; and of the man Akenside there were numerous subdivisions. Remarkable as a poet, he was even yet more noteworthy as a private individual in his extreme inconsistency." "Like Byron, he was lame, one of his legs being shorter than the other; and of this personal disfigurement he was even more sensitive than was the author of 'Childe Harold' of his deformity." "His father was a butcher of Newcastle-upon-Tyne, and one of his cleavers, falling from the shop-block, had irremediably injured the poet's foot when he was still a little child."

Akenside was three years at Edinburgh, a member of the Medical Society, and an industrious student; he then proceeded to Leyden, where he remained for some length of time and took his degree of doctor of physic, May 16, 1744. Although well versed in medicine and even advancing it somewhat in its scientific relations, he was never very successful in gaining clientage. As a poet he has won enduring fame by his "Pleasures of Imagination." As a medical writer he is favorably known by his treatise on dysentery.

To the names already given of physicians who were poets we should add those of Armstrong, author of "The Art of Health," Goldsmith, Smollet, Moore, the author of "Zeluco" and father of Sir John Moore, Aikin, Darwin, Mason Good, Wolcot, better known as Peter Pindar, Moir, and Crabbe. At home starts up in the minds of all Oliver Wendell Holmes, and less known, but of no mean merit, Percival.

Of pedagogues turned doctors, we read the names of John Bond, M.A., the learned commentator on Horace and Persius; next, of James Jurin, who from the position of a provincial schoolmaster raised himself to be regarded as first of the London physicians, and conspicuous among the philosophers of Europe. Why Arbuthnot is mentioned by Mr. Jeaffreson in this paper does not appear. Arbuthnot, the companion of Swift, the friend of Pope, and Parnell, and Garth, and Gay, and the life of the Kit-Kat Club; and last, and not least for his worldly prosperity, physician for some time to Queen Anne. To all this was the sad drawback, so common at that day, of habits of intemperance, and terrible suffering from asthma and melancholy.

We must not pass over in silence the chapter given to the lively and lucky and benevolent Quaker doctor, John Coakley Lettsom, by birth a West Indian, and for a time the pupil of Akenside the poet. He first began the practice of his profession in Tortola, where, during a residence of only five months, he earned the sum of £2000, ($10,000.)

"Ambitious of achieving a high professional position, he returned to Europe, visited the medical schools of Paris and Edinburgh, took his degree of M.D. at Leyden on the 29th of June, 1769. He was admitted a licentiate of the Royal College of Physicians of London in the same year, and in 1770 was elected a fellow of the College of Antiquaries. From this period till his death, in 1815, (November 20th,) he was one of the most prominent figures in the scientific world of London."

Lettsom had no high reputation for professional acquirements, and was wanting in a good preliminary education, but yet his income was larger than that of any other physician at the same time; in some years his receipts were as much as £12,000 ($60,000) a year. Although he pocketed such large sums, half his labors were entirely gratuitous. To give the mere list of his benevolent services would be to write a book about them. His tastes were various and refined: he liked pictures and works of sculpture, and spent large sums upon them, and he disbursed a fortune in surrounding himself at Camberwell with plants from the tropics. Room is not given to us to insert here some of the incidents characteristic of his goodness of heart and large practical benevolence. Of his numerous works the one most known to the general reader is the "History of Some of the Effects of Hard Drinking." It concludes with a scale of Temperance and Intemperance, in imitation of a thermometer. The vices, diseases, and punishments of the different stages of intemperance follow in tabular order.

We must now bring our notice of Mr. Jeaffreson's attractive work to a close, leaving untouched his chapters on "A Few More Quacks," "St. John Long," "The Quarrels of Physicians" and "The Loves of Physicians," "Medical Buildings," and "The Country Medical Man," together with a *hors d'œuvre*, entitled "Number Eleven—A Hospital Story." Our readers who are intent on making additions to the store with which we have furnished them must draw from the "Book" itself,—and we would commend them for doing so.

